# Validated registry of pre-dialysis chronic kidney disease: description of a large cohort

**DOI:** 10.1590/2175-8239-JBN-3841

**Published:** 2018-06-07

**Authors:** Rosalia Maria Nunes Henriques Huaira, Rogerio Baumgratz de Paula, Marcus Gomes Bastos, Fernando Antonio Basile Colugnati, Natália Maria da Silva Fernandes

**Affiliations:** 1 Universidade Federal de Juiz de Fora, Núcleo Interdisciplinar de Ensino e Pesquisas em Nefrologia, Juiz de Fora, MG, Brasil.

**Keywords:** Kidney Diseases, Diseases Registries, Epidemiology, Nefropatias, Registros de Doenças, Epidemiologia

## Abstract

**Introduction:**

Chronic diseases account for the majority of deaths in Brazil. These include hypertension (SAH) and *diabetes mellitus* (DM), which are the main causes of chronic kidney disease (CKD).

**Objective:**

This study aimed to validate the data of an electronic health record and to point out characteristics of the profile of these users in relation to clinical quality indicators for a pre-dialytic CKD.

**Methods:**

Retrospective cohort, August/2010 to December/2014. Included users > 18 years, with at least two queries. Variables analyzed: sociodemographic, underlying disease, main medications and main clinical indicators of control. A descriptive analysis was performed and the percentage of users was evaluated in the goals at admission and at the end of the study.

**Results:**

Exported, converted and validated data of 1,977 users with average follow-up time of 21 months. Of these, 51.4% were men, 58% were > 64 years of age and 81.6% were overweight. The main medications in use were diuretics (82.9%), BRAT (62%), Statin (60.7%) and ACE inhibitors (49.9%). The percentage of users with a decline in the glomerular filtration rate was 33.7%. Regarding glycated hemoglobin, users with CKD and DM, 36% were within the initial goal and 52.1% of the final. Blood pressure was at the target for admission at 34.3% and 49.8% at the end of follow-up.

**Conclusion:**

Validated data are of vital importance for health managers to monitor users. The population of this study is predominantly elderly, obese, requiring multi-professional care to slow the progression of the disease and decrease morbidity and mortality.

## RESUMO

**Introdução:**

As doenças crônicas são responsáveis pela maioria dos óbitos no Brasil. Estas incluem hipertensão (HAS) e *diabetes mellitus* (DM), que figuram como as principais causas de doença renal crônica (DRC).

**Objetivo:**

Este estudo teve como objetivo validar os dados de um sistema de prontuário eletrônico e apontar características do perfil dos usuários em relação aos indicadores clínicos de qualidade para DRC pré-dialítica.

**Métodos:**

Estudo observacional retrospectivo cobrindo o período de agosto de 2010 a dezembro de 2014. Foram incluídos indivíduos maiores de 18 anos, com pelo menos duas buscas. Variáveis analisadas: sociodemográficas, doença de base, principais medicamentos e principais indicadores clínicos de controle. Foi realizada uma análise descritiva e avaliado o percentual de usuários dentro das metas na internação e ao final do estudo.

**Resultados:**

Foram exportados, convertidos e validados os dados de 1.977 usuários com tempo médio de seguimento de 21 meses. Destes, 51,4% eram homens, 58% tinham idade superior a 64 anos e 81,6% apresentavam sobrepeso. Os principais medicamentos em uso foram diuréticos (82,9%), BRAT (62%), estatina (60,7%) e inibidores da ECA (49,9%). O percentual de usuários com queda da taxa de filtração glomerular foi de 33,7%. Em relação à hemoglobina glicada, dos usuários com DRC e DM, 36% estavam dentro da meta inicial e 52,1% da final. A pressão arterial estava dentro do alvo em 34,3% dos pacientes na internação e em 49,8% ao final do seguimento.

**Conclusão:**

Dados validados são de vital importância para os gestores da saúde monitorarem os usuários. A população deste estudo é predominantemente idosa, obesa, e com necessidade de cuidados multiprofissionais para retardar a progressão da doença e diminuir a morbimortalidade.

## INTRODUCTION

Noncommunicable diseases (NCDs) pose a great risk to the health of the Brazilian population. In 2009, NCDs accounted for 72.4% of all deaths and were ranked as the top cause of death in the nation.^1^ Though not officially included in this group of diseases, the footprint of chronic kidney disease (CKD) cannot be neglected, since its main causes are systemic hypertension (SH) and *diabetes mellitus* (DM), two of the most relevant NCDs.

The latest survey carried out by the Brazilian Society of Nephrology revealed that an estimated 112,004 patients with CKD were on dialysis in 2014.^2^ This number has grown by five percent annually since 2011, showing that CKD is a public health issue that demands early diagnosis and treatment.^3^

Registries play a key role in the improvement of preventive care and therapies, and help decrease the overall cost and enhance the effectiveness of healthcare. The evolution of CKD is well known, as are the interventions needed to delay the progression of the disease and prevent early death and the precocious start of renal replacement therapy (RRT).

According to the Federal Board of Medicine (CFM) and the Brazilian Society for Healthcare Information Technology (SBIS), electronic health registries are repositories of information on the health of individuals that can be managed electronically.^4^ These registries are regulated by Ordinance 2073 published in August of 2011 by the Ministry of Health.^5^

Numerous registries covering patients with CKD on dialysis are available in the USA,^6^^,^ 7 Europe,^8^^,^ 9 ^,^ 10 Australia,^11^^,^ 12 and Asia.^13^ Fewer registries are found in the Middle East,^14^^,^ 15 Latin America, and the Caribbean.^16^^,^ 17 In a systematic review, Liu *et al*.^18^ found 144 renal registries and analyzed 48 based on the following criteria: accessibility; and assessment of user, treatment, and outcome data. The authors concluded that only 17 registries offered good accessibility to general information.

Few registries follow patients with pre-dialysis CKD.^6^^-^ 17 Therefore, the electronic records used to characterize these individuals have to be validated in light of clinical quality indicators, for purposes of research and clinical/managerial decision-making.

This study aimed to validate the data stored in the registry of Centro Hiperdia Juiz de Fora and extract the characteristics of this group of patients in terms of clinical quality indicators for CKD.

## METHODS

The electronic health registry developed for Centro Hiperdia Juiz de Fora is based on an SQL database written in PHP Ajax - Javascript language. The records in the registry system were exported on CSV or Excel format and converted for use on SPSS 18.0. Validation was carried out using the syntax tool on SPSS to identify data inconsistencies and have them corrected or eliminated. Data validation - a condition required to ensure information reliability - safeguards the integrity and reliability of the information saved onto the database. The procedure eliminates incongruent patient information entered into the system by healthcare center staff.^19^

Data validation is about "correcting or improving data issues, missing values, inaccurate values or data off the expected range, answers not matching other answers saved onto the database, and eliminating repeated patient records".^20^ It is important to differentiate data validation from the validation of an instrument, defined as "making adjustments between the studied phenomenon and the theoretical concept to be measured".^21^

Many of the information entered into the Centro Hiperdia registry had inconsistencies and lack of standardization. The data had to be standardized before they were used in clinical research.

Patient names were the first items verified in the system. Healthcare center staff occasionally opened more than one record for the same patient. A verification was performed to check whether the repeated records belonged to namesakes or if two or more records had been opened for the same individual. When two or more records were found for one person, they were merged into one record containing all the information in the repeated records.

The next verification included the confirmation and standardization of information on age, sex, basic healthcare unit of origin, and city. The following data were also standardized: body weight, height, and individual/household income. The codes used to identify cities were changed to comply with the codes set out by the Brazilian Institute of Geography and Statistics (IBGE). Standardization allows data to be populated using a consistent format, so they can be used to compare between before and after care situations.

One of the stages of the verification revolved around confirming whether lab test results had been entered accurately into the system. Test results had all been entered into one single data field, thus precluding the identification of isolated test results. Test result entries did not follow a standard and had many inconsistencies; many missed percent signs and units, while others used different units - sometimes milligrams, sometimes grams - to describe the same variable. All entries had to be read, converted, and confirmed.

After the entries were standardized, they were checked for correctness. Faulty entries (repeated entries, entries with wrong data, data entered in the wrong fields etc.) were removed so that only valid data were used in clinical research.

This longitudinal retrospective cohort study enrolled patients seen between August of 2010 and December of 2014 at Centro Hiperdia de Juiz de Fora, Minas Gerais, Brazil, a clinic opened in 2010 by the Minas Gerais State Secretary of Health to monitor patients with SH, DM, and CKD. Since 2002, patients with CKD had been seen at the Minas Gerais Institute for Education and Research in Nephrology (IMEPEN), an institution created by teachers from the Medical School of the Federal University of Juiz de Fora to offer multi-professional care to individuals with CKD and delay the progression of the disease.

Centro Hiperdia de Juiz de Fora covers the following IBGE micro regions: Juiz de Fora (25 cities), Santos Dumont (3 cities), and São João Nepomuceno (9 cities), with a combined population of 837,991 individuals. Centro Hiperdia offers services to patients referred from the primary care clinics located in the cited micro regions. Demographic information is collected at the time of admission, while other variables are collected as patients are provided the care they need. Patients diagnosed with systemic hypertension at Centro Hiperdia met the following criteria: no response to three or more antihypertensive drugs used concurrently at pharmacologically effective dosages; target-organ lesions or suspicion of secondary hypertension. The criteria for DM were: patients with DM1 or DM2 on adequate metabolic control. The following criteria were applied to CKD: annual decreases in the glomerular filtration rate as calculated by the MDRD (Modification of Diet in Renal Disease) formula (∆eGFR) *≥* 5mL/min/year (baseline eGFR - end eGFR / number of months of observation x 12) mL/min/1.73m^2^ or proteinuria > 1.0 g/day or proteinuria < 1.0 g/day associated with hematuria or CKD stages 3B, 4 or 5 or abrupt increases ≥ 30% in serum creatinine levels or a 25% decrease in the estimated glomerular filtration rate after the start of therapy with renin-angiotensin-aldosterone system inhibitors.

The records of patients aged 18 years or older with at least two medical appointments followed at the CKD unit were included in the study. The following variables were analyzed: a) demographic variables: sex, age, skin color, city of origin, schooling, income, smoking, and drinking; b) clinical variables: blood pressure, weight, height, and the body mass index (BMI); c) workup variables: serum creatinine, estimated glomerular filtration rate (MDRD), fasting glucose, triglycerides, hemoglobin and glycated hemoglobin, total cholesterol, HDL and LDL, total calcium, phosphorus and potassium; d) medication: angiotensin-converting-enzyme (ACE) inhibitors, angiotensin II type 1 receptor blockers (ARBs), beta blockers, statins, acetylsalicylic acid (ASA), diuretics, insulin, biguanides, sulfonylureas, and fibrates; e) other variables: time on follow-up and number of medical appointments.

The project was approved by the Ethics Committee of the Federal University of Juiz de Fora and granted permit 36345514.1.0000.5139.

## RESULTS

**Figure 1:**
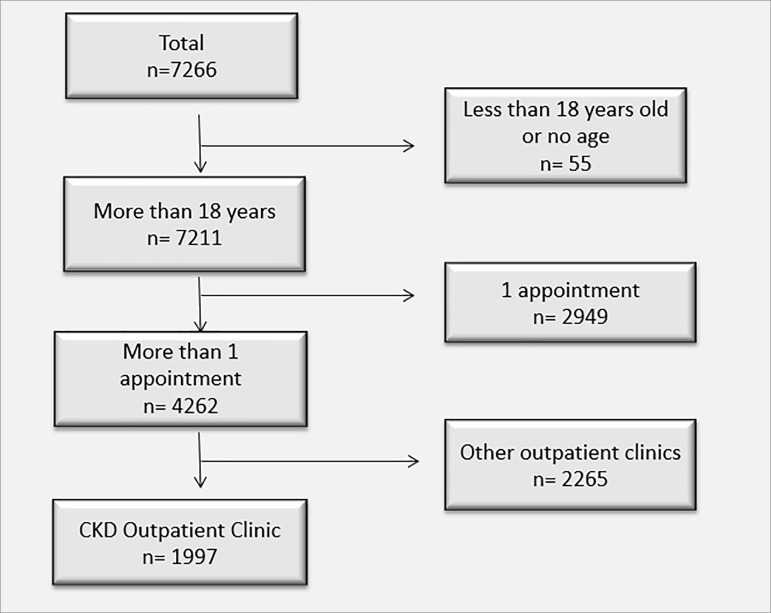
Flowchart describing the patient selection process.

Between August of 2010 and December of 2014, 7,266 patients were seen at Centro Hiperdia de Juiz de Fora. Fifty-five individuals (0.76%) were excluded for being under the age of 18 or for not having their ages stated in their records. Another 2,949 (40.5%) patients were excluded for having attended only one medical appointment; and 2,265 (31.2%) were not seen at the CKD outpatient unit. Therefore, 1,977 patients were included in the study (Figure 1).

A flag was included to validate the data entries in the system and block the acceptance of alphanumerical entries. Additional validation was performed to eliminate discrepant values. The results of 41 test types were validated. Table 1 shows the tests analyzed for the population enrolled in the study. The number of test results deemed not valid was low; only total calcium had more than one percent (1.36%) of its results categorized as not valid. The rate of rejected results was low for the other tests. Nearly all patients (98.1% - the highest rate among all tests) were tested for serum creatinine, whereas only 55.8% were tested for LDL cholesterol (the lowest rate among all tests).

**Table 1:** Proportion of tests categorized as not valid and frequency of tests per patient seen at the CKD unit of Centro Hiperdia Juiz de Fora (Aug 2010/Dec 2014)

Test type	% not valid	Patients tested	%
Creatinine	0.23%	1960	98.1%
Fasting glucose	0.01%	1934	96.8%
Triglycerides	0.12%	1896	94.9%
Hemoglobin	0.30%	1925	96.4%
Glycated hemoglobin	0.15%	1460	73.1%
Total calcium	1.36%	1651	82.7%
Potassium	0.24%	1911	95.7%
Phosphorus	0.21%	1562	78.2%
Total cholesterol	0.06%	1914	95.8%
HDL cholesterol	0.80%	1894	94.8%
LDL cholesterol	0.08%	1114	55.8%

Table 2 shows the demographic characterization of the included population. The patients had a mean age of 66.2 (± 13.4) years, with ages ranging from 19 and 102 years; 64% of them were 64 years or older. There were more men (51.4%) and individuals of white skin color (40.3%) with incomplete basic education (61.5%). Drinking alcohol was a habit for 15.1% of the participants, and 10% were smokers. Most of them lived in Juiz de Fora (75.5%). The mean individual monthly income of the studied group was 1.35 (± 1.48) minimum wages. A mean of 2.9 (± 1.55) individuals lived in each household.

**Table 2:** Sociodemographic description of the population served at the CKD unit of Centro Hiperdia Juiz de Fora (Aug 2010/Dec 2014)

Variable	n	%
Sex		
Male	1026	51.4%
Female	970	48.6%
Mean age		66.2 ± 13.4 anos
Skin color		
White	805	40.3%
Brown	686	34.4%
Black	501	25.1%
Yellow	5	0.3%
Schooling		
Illiterate	223	11.3%
Literate	55	2.8%
Incomplete basic education	1229	62.1%
Complete basic education	174	8.8%
Incomplete middle school	79	4.0%
Complete middle school	175	8.8%
Incomplete higher education	20	1.0%
Complete higher education	25	1.3%
Alcohol drinking	302	15.1%
Smoking	199	10.0%
Mean income. minimum wages		1.35 ± 1.48
Mean number of people residing in the household		2.9 ± 1.55 people
City		
Juiz de Fora	1506	75.5%
Other cities	488	24.5%

In table 3, the patients were followed for a mean of 21 (± 15) months at Centro Hiperdia. The mean number of medical appointments was 5.5 (± 4); the mean number of visits to the CKD unit was 3.2 (± 2.51). According to the BMI, nearly three quarters (75.4%) of the patients were overweight or obese. The patients were also followed in other healthcare units on account of DM (37.1%) and SH (37.8%). The more commonly seen stages of CKD were 3a (26.2%) and 3b (25.4%). The more frequently prescribed medications were diuretics (82.9%), ARBs (62%), and statins (60.7%). Concomitant administration of ACEi and ARBs was observed in 24% of the patients; the proportion went up to 30.5% when individuals with diabetes alone were analyzed. The use of ACEi per stage of CKD was as follows: 39.4% among individuals with stage 1 disease; 46.8% in stage 2 disease; 52.6% in stage 3A disease; 51.7% in stage 3B disease; 50.7% in stage 4 disease; and 46.1% in stage 5 disease. The following proportions were observed for patients on ARBs: 54.5% among individuals with stage 1 disease; 59.6% in stage 2 disease; 60.6% in stage 3A disease; 61.6% in stage 3B disease; 68.2% in stage 4 disease; and 69.7% in stage 5 disease.

**Table 3:** Description of the population served at the CKD unit of Centro Hiperdia Juiz de Fora in terms of clinical follow-up indicators (Aug 2010/Dec 2014)

Variable	N	%
Mean time on follow-up		21 ± 15 meses
Number of medical appointments at outpatient units		5.5 ± 4
Number of medical appointments at the CKD unit		3.2 ± 2.51
BMI categories		
Below 17 (very low weight)	1	0.1%
Between 17 and 18 (low weight)	23	1.2%
Between 18.5 and 24.99 (normal weight)	459	23.4%
Between 25 and 29.99 (overweight)	726	37.0%
Between 30 and 34.99 (obesity I)	456	23.3%
Between 35 and 39.99 (obesity II - severe)	176	9.0%
Above 40 (obesity III - morbid)	119	6.1%
Followed in other outpatient units		
*Diabetes mellitus*	755	37.8%
Hypertension	741	37.1%
CKD classification		
> = 90	66	3.4%
60 - 89	282	14.4%
45 - 59	523	26.7%
30 - 44	631	32.2%
15 - 29	381	19.4%
< 15	76	3.9%
Main medications		
ACEi	996	49.9%
ARBs	1239	62.0%
Beta blockers	1020	51.1%
Statins	1213	60.7%
ASA	937	46.9%
Diuretics	1656	82.9%
Biguanides	678	34.0%
Sulfonylureas	467	23.4%
Fibrates	210	10.5%
Insulin	145	7.3%
ACEi + ARBs	479	24%
Mean number of medications per patient		4.3 ± 1.9

Table 4 shows that the main workup parameters were under control, with the exception of triglyceride levels. Diabetic patients were analyzed separately and were found to have a mean fasting blood sugar level of 159.23 mg/dL *versus* 105.05 mg/dL of non-diabetic individuals.

**Table 4:** Description of the main test results of the patients seen at the CKD unit at Centro Hiperdia Juiz de Fora (Aug 2010/Dec 2014)

Test (mean value±sd)	Total	Non-diabetic patients	Diabetic patients
Creatinine (mg/dL)	1.76 ±0.98	1.78 ± 0.87	1.71±1.14
Hemoglobin (g/L)	13.18±1.95	13.33±1.93	12.94±1.96
Total calcium (mg/dL)	9.60 ± 0.91	9.61 ± 0.92	9.58 ± 0.90
HDL cholesterol (mg/dL)	47.05 ±13.28	47.35 ±13.20	46.57 ±13.41
LDL cholesterol (mg/dL)	115.90 ±44.95	117.35 ±43.29	113.86 ±47.16
Total cholesterol (mg/dL)	195.64 ±53.57	195.69 ± 50.95	195.57 ±57.51
Triglycerides (mg/dL)	174.11 ± 134.87	159.58 ±109.41	196.96 ± 164.76
Potassium (mEq/L)	4.68 ± 0.64	4.66 ± 0.62	4.70 ± 0.68
Phosphorus (mg/dL)	3.83 ± 0.98	3.73 ±0.98	3.99 ± 0.94
Fasting glucose (mg/dL)	125.92 ±64.80	105.05 ±37.85	159.23 ± 82.55
Glycated hemoglobin (%)	7.62 ±2.40	6.56 ±1.67	8.67 ± 2.56

Glycated hemoglobin levels of non-diabetic patients were on target in 81.7% of the cases at the start of the study and in 85.6% of the cases at the end of the study; 36% of the diabetic individuals had their glycated hemoglobin levels on target at baseline and 52.1% at the end of the study. In terms of blood pressure, 34.3% of the patients were on target at the start of the study and 49.8% at the end. The analysis of clinical quality indicators of CKD revealed that 33.7% of the sample was off target, with a mean eGFR decrease of 0.09 mL/min/1.73m^2^ and a median of zero. In order to correct it for time on follow-up, the annual decrease rate was calculated [(Delta eGFR/months on follow-up) *12], yielding a mean value of 4.94 ± 2.95 mL/min/1.73m^2^ and a median of 0.11mL/min/1.73m^2^. Proteinuria was cited in different shapes and forms, such as the albumin/creatinine ratio and 24-hour urinary protein, with units recorded in milligrams and grams. No clinical or statistically relevant changes were seen, and the variable did not follow a normal distribution (Table 5).

**Table 5:** Description of the population served at the CKD unit of Centro Hiperdia Juiz de Fora in terms of clinical quality indicators (Aug 2010/Dec 2014)

Variable	%
Decrease in glomerular filtration rate	33.7%
Annual decrease rate (mL/min/1.73m^2^)	4.94 ± 2.95
24-hour urine protein (mg/24h) (mean ± SD and median)	
Baseline	540 ± 1503 (153)
End	630 ± 1180 (171)
On target for glycated hemoglobin - diabetic patients	
Baseline	36%
End	52.1%
On target for glycated hemoglobin - non-diabetic patients	
Baseline	81.7%
End	85.6%
On target for blood pressure	
Baseline	34.3%
End	49.8%

Note: targets: glycated hemoglobin: < 7 (65 years of age) and < 8 (+65 years of age); blood pressure: 140/90 mmHg; GFR: < 5ml/year

## DISCUSSION

This study validated the entries in the electronic registry managed by Centro Hiperdia Juiz de Fora and characterized patient profiles in terms of clinical quality indicators used to monitor the progression of CKD. The studied population was predominantly elderly and obese and was under good workup and clinical management.

In the USA, the expenditure with end-stage renal disease amounted to USD 7 billion in 1991 and USD 30 billion in 2008.22 A study carried out in Brazil in 2008 revealed that the expenses incurred in by the Ministry of Health with high-cost procedures in the 2000-2004 period added up to BRL 8.6 billion, BRL 780 million of which with medication for CKD patients.^23^ It should be realized that despite the high costs involved in the care of CKD, there is extensive literature on the various outcomes of end-stage renal disease. However, even countries with large CKD registries such as the USA with the United States Renal Data System (USDRS) lack data on the pre-dialysis stage of the disease. Our study validated a registry of patients with pre-dialysis CKD seen in a healthcare center that serves individuals in southeast Minas Gerais, Brazil. The data may be used to better understand the priorities that have to be considered for this population.

A comparison between the study and the general Brazilian population revealed that although the proportion of individuals with ages greater than 60 years in the nation is 13.7%, in the study 58% of the patients were 64 or older.^24^ This is not a surprising finding, since this is a non-degenerative chronic illness. The Brazilian dialysis census reported that 32.5% of the patients were 64 or older.^2^ The 2011 census indicated that women accounted for 51.6% of the Brazilian population, revealing a slight difference from our study and from the dialysis census, in which 48.6% and 42% of the individuals were females, respectively. From the standpoint of race or skin color, our data were in agreement with the proportions seen in the Brazilian population, with greater percentages of people of brown/black skin color. In terms of schooling, most of the individuals in the registry had incomplete basic education. This finding differed from the level of schooling of the Brazilian population aged 25 or older, possibly due to the greater mean age of the individuals in the registry. For the same reason, the mean income of the individuals in the registry (1.35 minimum wages) was lower than the mean income of the Brazilian population (2.47 minimum wages).^25^

The proportion of smokers was similar to the percentage reported in a previous study carried out at Centro Hiperdia de Juiz de Fora^26^ at 10.12%. This value is lower than the rate published by Vigitel (17%) in 2008,^27^ and reflects the countrywide strategy of providing counseling to smokers.^26^

Obesity is a global epidemics that affected 17.9% of the Brazilian population in 2014 according to Vigitel, with adult individuals living in the Brazilian Southeast accounting for the higher proportion of overweight subjects, with 50.45%.^27^ The most recent Brazilian Dialysis Census found that 37% of the patients were overweight, obese or morbidly obese,^2^ a proportion lower than the one found in our study (75.4%), showing that more aggressive approaches need to be implemented to tackle this preventable risk factor affecting a number of non-degenerative chronic illnesses.

A study carried out by our research group reported a prevalence of 17% of diabetic nephropathy.^25^^,^ 28 However, a prevalence of 37.8% of *diabetes mellitus* was observed in the present study. This data shows that the number of diabetic individuals has grown as a result of factors such as population growth and aging, increased urbanization, growing prevalence of obesity and sedentarism, and the longer survival of individuals with DM. Quantifying the current prevalence of DM and estimating the number of people with diabetes in the future is a relevant exercise, since it allows for better planning and rational allocation of resources to address the issue.^29^ An epidemics of DM is in course, with forecasts indicating that 300 million individuals will be suffering from the disease in 2030. About two thirds of the individuals with DM live in developing countries, where the epidemics has been more intense, with growing numbers of affected individuals at younger ages, coexisting with infectious diseases and the burden they entail.^29^ Although elevated, the prevalence of diabetes reported in our study might have been underestimated, since the mean fasting blood sugar level of non-diabetic individuals was 105 mg/dL.

Population studies performed in Brazilian cities within the last 20 years have described prevalence rates of systemic hypertension above 30%. Considering BP values ≥ 140/90 mmHg, 22 studies reported prevalence rates ranging between 22.3% and 43.9% (mean value: 32.5%), above 50% for individuals aged 60-69 years, and of 75% for subjects aged 70 or older.^29^^,^ 30 BP management has improved significantly in Canada, moving from 13.2% of the patients on target in 1992 to 64.6% in 2007.^31^ Better BP management has been associated with improved cardiovascular outcomes, as recently described by Xie.^32^ In our study, 34.3% of the patients were on target for BP at baseline *versus* 49.8% at the end of the study.

In terms of use of medication, concomitant use of ACEi and ARBs has precise indications such as difficult-to-treat proteinuria and congestive heart failure.^33^ The proportion of patients using one of or the two drugs was low in our study. The use of ACEi decreased among subjects with CKD stages 1 and 5, while ARBs were more used by individuals with CKD stages 4 and 5. A systematic review published by Catalá-Lopez found that the GFR of diabetic individuals taking both drugs did not deteriorate.^34^

GFR decline was subtle, suggesting the patients were being well managed. The mean change in the GFR was positive by 5 ml, i.e., renal function improved, reflecting the quality and good outcomes of a proper multi-professional healthcare model. The same rationale applies to the significant improvement observed in glycated hemoglobin in diabetic patients.

Given the limitations of the study, not all tests ordered by the attending physicians were carried out. In addition, the tests were not run in the same laboratory. This "real-life" study shed light on the troubles experienced by Brazilian physicians with getting tests done.

## CONCLUSION

Validated data are of vital importance for healthcare managers to monitor patient populations. Old age, low income, obesity, polypharmacy, and little educational qualification are traits common to most of the individuals in our registry. They make up a vulnerable population in need of intensive multi-professional care to delay the progression of the disease and decrease morbidity and mortality. The positive delta seen in the glomerular filtration rate supports the fact that the main goal is being reached: to delay the start of renal replacement therapy and thus improve patient quality-of-life and reduce care costs.

## References

[1] Schmidt MI, Duncan BB, Azevedo e Silva G, Menezes AM, Monteiro CA, Barreto SM (2011). Chronic non-communicable diseases in Brazil: burden and current
challenges. Lancet.

[2] Sesso RC, Lopes AA, Thomé FS, Lugon JR, Martins CT (2016). Brazilian Chronic Dialysis Census 2014. J Bras Nefrol.

[3] Bastos MG, Kirsztajn GM (2011). Chronic kidney disease: importance of early diagnosis, immediate
referral and structured interdisciplinary approach to improve outcomes in
patients not yet on dialysis. J Bras Nefrol.

[4] CFM, SBIS (2012). Cartilha sobre Prontuário Eletrônico. A certificação de Sistemas de
Registro Eletrônico de Saúde.

[5] Brasil, Ministério da Saúde (2011). Portaria Nº 2073, de 31 de agosto de 2011. Regulamenta o uso de padrões
de interoperabilidade e informação em saúde para sistemas de informação em
saúde no âmbito do Sistema Único de Saúde, nos níveis Municipal, Distrital,
Estadual e Federal, e para os sistemas privados e do setor de saúde
suplementar.

[6] Boulware LE, Tangri N, Ephraim PL, Scialla JJ, Sozio SM, Crews DC, DEcIDE ESRD Patient Outcomes in Renal Disease Study
Investigators (2012). Comparative effectiveness studies to improve clinical outcomes in
end stage renal disease: the DEcIDE patient outcomes in end stage renal
disease study. BMC Nephrol.

[7] Powe NR, Tarver-Carr ME, Eberhardt MS, Brancati FL (2003). Receipt of renal replacement therapy in the United States: a
population-based study of sociodemographic disparities from the Second
National Health and Nutrition Examination Survey (NHANES II). Am J Kidney Dis.

[8] Couchoud C, Dantony E, Elsensohn MH, Villar E, Ecochard R, REIN Registry (2013). Modelling treatment trajectories to optimize the organization of
renal replacement therapy and public health decision-making. Nephrol Dial Transplant.

[9] Couchoud C, Lassalle M, Stengel B, Jacquelinet C (2009). [Renal Epidemiology and Information Network: 2007 annual
report]. Nephrol Ther.

[10] Spithoven EM, Kramer A, Meijer E, Orskov B, Wanner C, Abad JM, ERA-EDTA Registry, EuroCYST Consortium, WGIKD (2014). Renal replacement therapy for autosomal dominant polycystic
kidney disease (ADPKD) in Europe: prevalence and survival--an analysis of
data from the ERA-EDTA Registry. Nephrol Dial Transplant.

[11] Gray NA, Mahadevan K, Campbell VK, Noble EP, Anstey CM (2013). Data quality of the Australia and New Zealand Dialysis and
Transplant Registry: a pilot audit. Nephrology.

[12] Venuthurupalli SK, Hoy WE, Healy HG, Salisbury A, Fassett RG, CKD.QLD group (2012). CKD.QLD: chronic kidney disease surveillance and research in
Queensland, Australia. Nephrol Dial Transplant.

[13] Rajapurkar MM, John GT, Kirpalani AL, Abraham G, Agarwal SK, Almeida AF (2012). What do we know about chronic kidney disease in India: first
report of the Indian CKD registry. BMC Nephrol.

[14] Aghighi M, Mahdavi-Mazdeh M, Zamyadi M, Heidary Rouchi A, Rajolani H, Nourozi S (2009). Changing epidemiology of end-stage renal disease in last 10 years
in Iran. Iran J Kidney Dis.

[15] Ajami S, Askarianzadeh M, Mortazavi M (2015). Developing a provisional and national renal disease registry for
Iran. J Res Med Sci.

[16] Soyibo AK, Barton EN (2009). Chronic renal failure from the English-speaking Caribbean: 2007
data. West Indian Med J.

[17] Soyibo AK, Barton EN (2007). Report from the Caribbean renal registry, 2006. West Indian Med J.

[18] Liu FX, Rutherford P, Smoyer-Tomic K, Prichard S, Laplante S (2015). A global overview of renal registries: a systematic
review. BMC Nephrol.

[19] Macoratti JC (2018). SilverLight - Fazendo a validação no databinding (C#).

[20] Gliklich RE, Dreyer NA (2014). Registries for evaluating patient outcomes: a user's guide.

[21] Monteiro GTR, da Hora HRM (2015). Pesquisa em Saúde Pública: como desenvolver e validar instrumentos de
coleta de dados.

[22] Navaneethan SD, Jolly SE, Schold JD, Arrigain S, Saupe W, Sharp J (2011). Development and validation of an electronic health record-based
chronic kidney disease registry. Clin J Am Soc Nephrol.

[23] Silva GD (2008). Avaliação dos gastos realizados pelo Ministério da Saúde com
medicamentos de alto custo utilizados no tratamento da DRC por pacientes do
SUS no estado de Minas Gerais - 2000 a 2004.

[24] Brasil, Instituto Brasileiro de Geografia e Estatística (2015). Síntese de indicadores sociais: uma análise das condições de vida da
população brasileira 2015.

[25] Tirapani Ldos S, Pinheiro HS, Mansur HN, Oliveira Dd, Huaira RM, Huaira CC (2015). Impact of social vulnerability on the outcomes of predialysis
chronic kidney disease patients in an interdisciplinary
center. J Bras Nefrol.

[26] Campos Tda S, Richter KP, Cupertino AP, Galil AG, Banhato EF, Colugnati FA (2014). Cigarette smoking among patients with chronic
diseases. Int J Cardiol.

[27] Brasil, Ministério da Saúde Vigilância de fatores de risco e proteção para doenças crônicas por
inquérito telefônico (VIGITEL).

[28] Pereira AC, Carminatti M, Fernandes NM, Tirapani Ldos S, Faria RS, Grincenkov FR (2012). Association between laboratory and clinical risk factors and
progression of the predialytic chronic kidney disease. J Bras Nefrol.

[29] Diretrizes da Sociedade Brasileira de Diabetes 2013-2014.

[30] Brandão AA, Magalhães MEC, Ávila A, Tavares A, Machado CA, Campana EMG (2010). Conceituação, epidemiologia e prevenção primária. Diretrizes
Brasileiras de Hipertensão VI. Capítulo 1. J Bras Nefrol.

[31] McAlister FA, Wilkins K, Joffres M, Leenen FH, Fodor G, Gee M (2011). Changes in the rates of awareness, treatment and control of
hypertension in Canada over the past two decades. CMAJ.

[32] Xie X, Atkins E, Lv J, Bennet A, Neal B, Ninomiya T (2016). Effects of intensive blood pressure lowering on cardiovascular
and renal outcomes: updated systematic review and
meta-analysis. Lancet.

[33] Fröhlich H, Nelges C, Täger T, Schwenger V, Cebola R, Schnorbach J (2016). Long-term changes of renal function in relation to ace
inhibitor/angiotensin receptor blocker dosing in patients with heart failure
and chronic kidney disease. Am Heart J.

[34] Catalá-López F, Macías Saint-Gerons D, González-Bermejo D, Rosano GM, Davis BR, Ridao M (2016). Cardiovascular and Renal Outcomes of Renin-Angiotensin System
Blockade in Adult Patients with Diabetes Mellitus: A Systematic Review with
Network Meta-Analyses. PLoS Med.

